# Solving acoustic scattering problems by the isogeometric boundary element method

**DOI:** 10.1007/s00366-024-02013-y

**Published:** 2024-07-01

**Authors:** Jürgen Dölz, Helmut Harbrecht, Michael Multerer

**Affiliations:** 1 Institute for Numerical Simulation, University of Bonn, Friedrich-Hirzebruch-Allee 7, 53115 Bonn, Germany; 2 Department of Mathematics and Computer Science, University of Basel, Spiegelgasse 1, 4051 Basel, Switzerland; 3 Istituto Eulero, Università della Svizzera italiana, Via la Santa 1, 6962 Lugano, Switzerland

**Keywords:** Boundary integral equation, Isogeometric analysis, Helmholtz equation, Scattering problem

## Abstract

We solve acoustic scattering problems by means of the isogeometric boundary integral equation method. In order to avoid spurious modes, we apply the combined field integral equations for either sound-hard scatterers or sound-soft scatterers. These integral equations are discretized by Galerkin’s method, which especially enables the mathematically correct regularization of the hypersingular integral operator. In order to circumvent densely populated system matrices, we employ the isogeometric embedded fast multipole method, which is based on interpolation of the kernel function under consideration on the reference domain, rather than in space. To overcome the prohibitive cost of the potential evaluation in case of many evaluation points, we also accelerate the potential evaluation by a fast multipole method which interpolates in space. The result is a frequency stable algorithm that scales essentially linear in the number of degrees of freedom and potential points. Numerical experiments are performed which show the feasibility and the performance of the approach.

## Introduction

Acoustic wave scattering appears in many places in engineering practice. This includes, for instance, the modeling of sonar and other methods of acoustic location, as well as outdoor noise propagation and control, especially stemming from automobiles, railways or aircrafts. Since an analytical solution of scattering problems is in general impossible, numerical approaches are called for the approximate solution.

Most acoustic scattering problems may be formulated in the frequency domain by employing the Helmholtz equation. Assume that an acoustic wave encounters an impenetrable, bounded obstacle $\varOmega \subset \mathbb {R}^3$, having a Lipschitz smooth boundary $\varGamma \mathrel {\mathrel {\mathop :}=}\partial \varOmega$, and, as a consequence, gets scattered. Given the *incident plane wave* $u_{\text {inc}}({\varvec{x}}) = e^{i\kappa \langle {\varvec{d}}, {\varvec{x}}\rangle }$ with known wavenumber $\kappa$ and direction ${\varvec{d}}$, where $\Vert {\varvec{d}}\Vert _2=1$, the goal is to compute the *scattered wave* $u_{\textrm{s}}$. The physical model behind this is as follows. The *total wave* $u = u_{\text {inc}}+u_{\text {s}}$ satisfies the exterior Helmholtz equation

1 $$\begin{aligned} \Delta u + \kappa ^2 u = 0\ \text {in}\ \mathbb {R}^3\setminus \overline{\varOmega }. \end{aligned}$$

The boundary condition at the scatterer’s surface depends on its physical properties. If the scatterer constitutes a *sound-soft* obstacle, then the acoustic pressure vanishes at $\varGamma$ and we have the homogeneous Dirichlet condition

2 $$\begin{aligned} u = 0\ \text {on}\ \varGamma . \end{aligned}$$

Whereas, if the scatterer constitutes a *sound-hard* obstacle, then the pressure gradient vanishes at $\varGamma$ in normal direction and we have the homogeneous Neumann condition

3 $$\begin{aligned} \frac{\partial u}{\partial \varvec{n}} = 0\ \text {on}\ \varGamma . \end{aligned}$$

The behavior towards infinity is imposed by the Sommerfeld radiation condition

4 $$\begin{aligned} \lim _{r\rightarrow \infty } r \left\{ \frac{\partial u_s}{\partial r} - i\kappa u_s\right\} = 0,\ \text {where}\ r\mathrel {\mathrel {\mathop :}=}\Vert \varvec{x}\Vert _2. \end{aligned}$$

It implies the asymptotic expansion

$$\begin{aligned} u_s(\varvec{x}) = \frac{e^{i\kappa \Vert \varvec{x}\Vert _2}}{\Vert \varvec{x}\Vert _2} \left\{ u_\infty \Big (\frac{\varvec{x}}{\Vert \varvec{x}\Vert _2}\Big ) + \mathcal {O}\Big (\frac{1}{\Vert \varvec{x}\Vert _2}\Big )\right\} \end{aligned}$$

as $\Vert \textbf{x}\Vert _2\rightarrow \infty$. Herein, the function

$$\begin{aligned} u_\infty :\mathbb {S}^1 \mathrel {\mathrel {\mathop :}=}\{\hat{\varvec{x}}\in \mathbb {R}^d:\Vert \hat{\varvec{x}}\Vert _2=1\}\rightarrow \mathbb {C} \end{aligned}$$

is called the *far-field pattern*, which is always analytic in accordance with [6, Chapter 6].

To avoid the discretization of the unbounded exterior domain $\mathbb {R}^3\setminus \overline{\varOmega }$, one can exploit the integral equation formalism to compute the numerical solution of acoustic scattering problems. Then, one arrives at a boundary integral equation only defined on the boundary $\varGamma$. We will employ here the methodology of *isogeometric analysis* (IGA) to discretize this boundary integral equation. IGA has been introduced in [22] in order to incorporate simulation techniques into the design workflow of industrial development. The goal is thus to unify the CAD representation of the scatterer with the boundary element discretization of the integral equation in terms of *non-uniform rational B-splines* (NURBS). We refer the reader to [8, 9, 13, 25, 29, 31, 34] and the references therein for details of the isogeometric boundary element method.

While a reformulation of the scattering problem by means of a boundary integral equation replaces the problem posed in the unbounded domain by a problem posed on the scatterer’s closed boundary, the underlying boundary integral operator and potential evaluation are non-local operators. This yields densely populated system matrices of the underlying linear systems of equations. Moreover, the potential evaluation also behaves like a dense matrix. Thus, in case of discretizations with many degrees of freedom and/or many potential evaluation points, the naive approach becomes computationally prohibitive. The fast multipole method (FMM) [19] aims to overcome these drawbacks by means of nested local low-rank approximations. While usually the kernel function under consideration is approximated in space, in the isogeomtric context it is preferable to follow the approach of [20] and to interpolate the kernel function on the reference domain to which we refer to as the *isogeometric embedded FMM* [13, 14]. Especially, this reduces the complexity from $\mathcal {O}(p^6)$ to $\mathcal {O}(p^4)$ in the FMM interpolation degree *p*.

The isogeometric embedded FMM has been developed in [13, 14] and was made accessible to the public by the software C++ library Bembel.^1^ [10, 11]. Bembel combines a Galerkin discretization with the fast multipole method to accelerate the computation of the far-field while reducing memory requirements. It has for example been applied successfully to engineering problems arising from electromagnetics [15, 23] or from acoustics [16]. It has also been used in other applications, for example, to optimize periodic structures [21], in uncertainty quantification [12, 16], in the coupling of FEM and BEM [17], or in the partial element equivalent circuit (PEEC) method [33].

The contribution of this article is to present an isogeometric, frequency stable algorithm for the solution of acoustic obstacle scattering problems with essentially linear complexity in the number of boundary elements and potential evaluation points. To this end, we use combined field integral equations to obtain frequency robust boundary integral formulations of sound-soft and sound-hard acoustic scattering problems. We demonstrate that the required hypersingular operator of the Helmholtz equation indeed fits into the framework of the isogeometric embedded FMM when discretized by means of the Galerkin scheme. This allows the efficient compression of the combined field integral equations by the isogeometric embedded FMM and thus their efficient solution. To overcome the non-locality of the potential evaluation, we apply an additional FMM to the potential evaluation operator to achieve an overall linearly scaling algorithm. Although we restrict ourselves to the sound-soft and sound-hard cases, the presented concepts are also suitable to treat penetrable obstacles, i.e. objects described by a different diffractive index to the free space.

The rest of this article is structured as follows. In Sect. 2, we introduce the frequency stable boundary integral equations which are employed to solve either sound-hard or sound-soft scattering problems. Section 3 recapitulates the basic concepts from isogeometric analysis and introduces the discretization spaces that will be used later on. In Sect. 4, we discuss the discretization of the required boundary integral operators. In particular, we address the regularization of the hypersingular operator. Moreover, we comment on the isogeometric fast multipole method for the fast assembly of the operators and the potential evaluation. The numerical experiments are presented in Sect. 5, where we first validate the implementation in case of the (smooth) torus and then consider a turbine blade geometry which consists of 120 patches. Finally, concluding remarks are stated in Sect. 6.

## Boundary integral equation method

In order to solve the boundary value problem (1)–(4), we shall employ a suitable reformulation by boundary integral equations. To this end, we introduce the acoustic single layer operator

$$\begin{aligned}\mathcal {V}:&H^{-1/2}(\varGamma )\rightarrow H^{1/2}(\varGamma ),\\& (\mathcal {V}\rho )({\varvec{x}}):=\int _{\varGamma } G({\varvec{x}},{\varvec{y}})\rho ({\varvec{y}}){\text {d}}\sigma _{\varvec{y}},\end{aligned}$$

the acoustic double layer operator

$$\begin{aligned}\mathcal {K}:&L^2(\varGamma )\rightarrow L^2(\varGamma ),\\& (\mathcal {K}\rho )({\varvec{x}}):=\int _{\varGamma } \frac{\partial G({\varvec{x}},{\varvec{y}})}{\partial {\varvec{n}}_{\varvec{y}}}\rho ({\varvec{y}}){\text {d}}\sigma _{\varvec{y}},\end{aligned}$$

its adjoint

$$\begin{aligned}\mathcal {K}^\star :&L^2(\varGamma )\rightarrow L^2(\varGamma ),\\& (\mathcal {K}^\star \rho )({\varvec{x}}):=\int _{\varGamma } \frac{\partial G({\varvec{x}},{\varvec{y}})}{\partial {\varvec{n}}_{\varvec{x}}}\rho ({\varvec{y}}){\text {d}}\sigma _\textbf{y},\end{aligned}$$

as well as the acoustic hypersingular operator

5 $$\begin{aligned}\mathcal {W}:&H^{1/2}(\varGamma )\rightarrow H^{-1/2}(\varGamma ),\\& (\mathcal {W}\rho )({\varvec{x}}):=-\frac{1}{\partial {\varvec{n}}_{\varvec{x}}}\int _{\varGamma } \frac{\partial G({\varvec{x}},{\varvec{y}})}{\partial {\varvec{n}}_{\varvec{y}}} \rho ({\varvec{y}}){\text {d}}\sigma _{\varvec{y}}.\end{aligned}$$

Here, ${\varvec{n}}_{\varvec{x}}$ and ${\varvec{n}}_{\varvec{y}}$ denote the outward pointing normal vectors at the surface points ${\varvec{x}}, {\varvec{y}}\in \varGamma$, respectively, while $G(\cdot ,\cdot )$ denotes the fundamental solution for the Helmholtz equation. In three spatial dimensions, the latter is given by

$$\begin{aligned} G({\varvec{x}}, {\varvec{y}}) = \frac{e^{i\kappa \Vert {\varvec{x}}-{\varvec{y}}\Vert _2}}{4\pi \Vert {\varvec{x}}-{\varvec{y}}\Vert _2}. \end{aligned}$$

Although the Helmholtz problem (1)–(4) is uniquely solvable, the respective boundary integral formulation might not if $\kappa ^2$ is an eigenvalue for the Laplacian inside the scatterer $\varOmega$. In order to avoid such *spurious modes*, we employ combined field integral equations in the following. Then, for some real $\eta \ne 0$, the solution of the boundary integral equation

6 $$\begin{aligned} \bigg (\frac{1}{2}+\mathcal {K}^\star -i\eta \mathcal {V}\bigg ) \frac{\partial u}{\partial {\varvec{n}}} = \frac{\partial u_{\text {inc}}}{\partial {\varvec{n}}} -i\eta u_{\text {inc}} \end{aligned}$$

gives rise to the scattered wave in accordance with

7 $$\begin{aligned} u_{\text {s}}({\varvec{x}}) = \int _\varGamma G({\varvec{x}},{\varvec{y}}) \frac{\partial u({\varvec{y}})}{\partial {\varvec{n}}_{\varvec{y}}}{\text {d}}\sigma _{\varvec{y}} \end{aligned}$$

in case of sound-soft scattering problems. In case of sound-hard obstacles, we will solve the integral equation

8 $$\begin{aligned} \bigg (\frac{1}{2} - \mathcal {K} + i\eta \mathcal {W}\bigg )u = u_{\text {inc}}-i\eta \frac{\partial u_{\text {inc}}}{\partial \varvec{n}}. \end{aligned}$$

Having solved (8), the scattered wave is computed by

9 $$\begin{aligned} u_{\text {s}}({\varvec{x}}) = \int _\varGamma \frac{\partial G({\varvec{x}},{\varvec{y}})}{\partial {\varvec{n}}_{\varvec{y}}}u({\varvec{y}}){\text {d}}\sigma _{\varvec{y}}. \end{aligned}$$

Notice that the boundary integral equations (6) and (8) are always uniquely solvable, independent of the wavenumber $\kappa$, see [5, 6, 24].

## Isogeometric analysis

### B-splines

We shall give a brief introduction to the basic concepts of isogeometric analysis, starting with the definition of the B-spline basis, followed by the description of the scatterer by using NURBS. To this end, let $\mathbb {K}$ be either $\mathbb {R}$ or $\mathbb {C}$. The original definitions (or equivalent notions) and proofs, as well as basic algorithms, can be found in most of the standard spline and isogeometric literature [7, 22, 27, 28, 32].

#### Definition 1

**Fig. 1:**
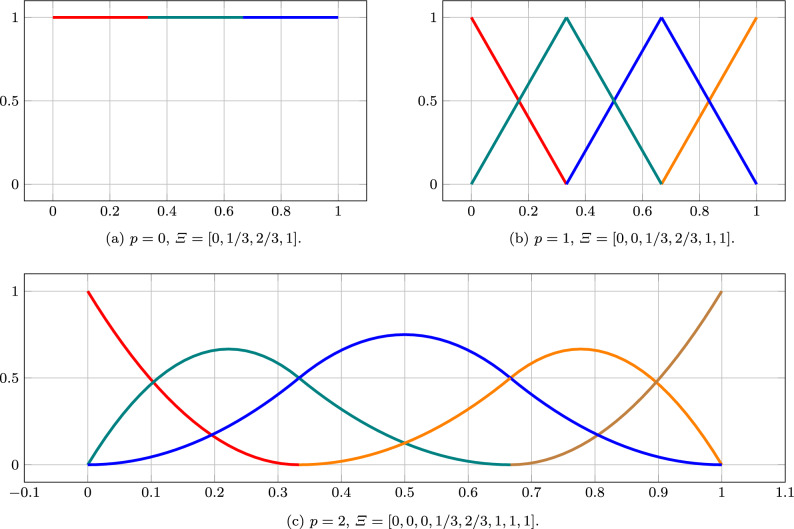
B-spline bases for $p=0,1,2$ and open knot vectors with interior knots 1/3 and 2/3

Let $0\le p\le k$. We define a *p-open knot vector* as a set

$$\begin{aligned}&\varXi = \big [\underbrace{\xi _0 = \cdots =\xi _{p}}_{=0}\le \cdots \\&\cdots \le \underbrace{\xi _{k}=\cdots =\xi _{k+p}}_{=1}\big ] \in [0,1]^{k+p+1}, \end{aligned}$$

where *k* denotes the number of control points. The associated basis functions are given by $\{b_j^p\}_{j=0}^{k-1}$ for $p=0$ as

$$\begin{aligned} b_j^0(x) =\left\{ \begin{array}{ll} 1, &{} \text {if }\xi _j\le x<\xi _{j+1}, \\ 0, &{} \text {otherwise},\end{array}\right. \end{aligned}$$

and for $p>0$ via the recursive relationship

$$\begin{aligned} b_j^p(x) = \frac{x-\xi _j}{\xi _{j+p}-\xi _j}b_j^{p-1}(x) +\frac{\xi _{j+p+1}-x}{\xi _{j+p+1}-\xi _{j+1}}b_{j+1}^{p-1}(x), \end{aligned}$$

see Fig. 1. A *spline* is then defined as a function

$$\begin{aligned} f(x) = \sum _{0\le j< k}p_jb_j^p(x), \end{aligned}$$

where $\{p_j\}_{j=0}^{k-1}\subset \mathbb {K}$ denotes the set of *control points*. If one sets $\{{\varvec{p}}_j\}_{j=0}^{k-1}\subset \mathbb {R}^d$, then *f* will be called a *spline curve*.

Having the spline functions at hand, we can introduce the spline spaces which serve as fundament for the definition of the ansatz and test spaces of the boundary element method. To keep the notation lightweight, we will ignore in the definition the dependence on the underlying field, which is either $\mathbb {R}$ or $\mathbb {C}$.

#### Definition 2

Let $\varXi$ be a *p*-open knot vector containing $k+p+1$ elements. We define the *spline space* $S_{p}(\varXi )$ as the space spanned by $\{b_j^p\}_{j=0}^{k-1}$.

Finally, we should consider the relation between the spline spaces and the underlying mesh relative to a certain mesh size.

#### Definition 3

For a knot vector $\varXi ,$ we define the *mesh size* *h* to be the maximal distance

10 $$\begin{aligned} h\mathrel {\mathrel {\mathop :}=}\max _{j=0}^{k+p-1}h_j,\quad \text {where}\quad h_j\mathrel {\mathrel {\mathop :}=}\xi _{j+1}-\xi _{j}, \end{aligned}$$

between neighboring knots. We call a knot vector *quasi uniform*, when there exists a constant $\theta \ge 1$ such that for all *j* the ratio $h_j\cdot h_{j+1}^{-1}$ satisfies $\theta ^{-1}\le h_j\cdot h_{j+1}^{-1} \le \theta .$

B-splines on higher dimensional domains are constructed through simple tensor product relationships for ${\varvec{p}}_{j_1,\dots j_\ell }\in \mathbb {K}^d$ via

11 $$\begin{aligned} \begin{aligned}&f(x_1,\dots ,x_\ell )\\&\quad =\sum _{j_1=0}^{k_1-1}\dots \sum _{j_\ell =0}^{k_\ell -1} {\varvec{p}}_{j_1,\dots ,j_\ell } \cdot b_{j_1}^{p_1}(x_1)\cdots b_{j_\ell }^{p_\ell }(x_\ell ), \end{aligned} \end{aligned}$$

which allows *tensor product spline spaces* to be defined as

$$\begin{aligned} S_{p_1,\dots ,p_\ell }(\varXi _1,\dots ,\varXi _\ell ). \end{aligned}$$

Throughout this article, we will reserve the letter *h* for the mesh size (10). All knot vectors will be assumed to be quasi uniform, such that the usual spline theory is applicable [1, 27, 28].

### Isogeometric representation of the scatterer

We assume that the boundary $\varGamma$ of the scatterer is closed and Lipschitz continuous. For the remainder of this article, we assume that it is given patchwise as $\varGamma =\bigcup _{j=1}^n\varGamma _j$, i.e. that it is induced by $C^\infty$-diffeomorphisms

12 $$\begin{aligned} \textbf{F}_j:\widehat{\varOmega } = [0,1]^2 \rightarrow \varGamma _j \subset \mathbb {R}^3. \end{aligned}$$

This regularity is required for the parametric fast multipole method employed later on.

In the spirit of isogeometric analysis, these mappings are given by NURBS mappings, i.e. by

$$\begin{aligned} \textbf{F}_j(x,y)\mathrel {\mathrel {\mathop :}=}\sum _{j_1=0}^{k_1-1}\sum _{j_2=0}^{k_2-1} \frac{c_{j_1,j_2} b_{j_1}^{p_1}(x) b_{j_2}^{p_2}(y) w_{j_1,j_2}}{\sum _{i_1=0}^{k_1-1}\sum _{i_2=0}^{k_2-1} b_{i_1}^{p_1}(x) b_{i_2}^{p_2}(y) w_{i_1,i_2}} \end{aligned}$$

with control points $c_{j_1,j_2}\in \mathbb {R}^3$ and weights $w_{i_1,i_2}>0$. We will moreover require that, for any interface $D = \varGamma _j\cap \varGamma _i \ne \emptyset$, the NURBS mappings coincide, i.e. that, up to rotation of the reference domain, one finds $\textbf{F}_j(\cdot ,1) \equiv \textbf{F}_i(\cdot ,0)$.

### Ansatz and test spaces

The mappings of (12) give rise to the transformations

$$\begin{aligned} \iota _j(f) \mathrel {\mathrel {\mathop :}=}f\circ \textbf{F}_j, \end{aligned}$$

which can be utilized to define discrete spaces patchwise, by mapping the space of tensor product B-splines as in (11) with

$$\begin{aligned} \varXi _{p,m} \mathrel {\mathrel {\mathop :}=}\big [ \underbrace{0,\dots ,0}_{p+1\text { times}}, {1}/{2^m},\dots , {(2^m-1)}/{2^m}, \underbrace{1,\dots ,1}_{p+1\text { times}}\big ] \end{aligned}$$

to the geometry. Here, the variable *m* denotes the level of uniform refinement. For the purposes of discretizing $\mathcal {V}$, $\mathcal {K}$, and $\mathcal {K}^\star$, the global function space on $\varGamma$ defined by

$$\begin{aligned}\mathbb {S}_{p,m}^2(\varGamma ) \mathrel {\mathrel {\mathop :}=}\Big \{& f\in H^{-1/2}(\varGamma ):f_{|\varGamma _j} \equiv \iota _j^{-1}(g)\\&\text { for some }g\in S_{p,p}(\varXi _{p,m},\varXi _{p,m})\Big \}, \end{aligned}$$

as commonly done in the isogeometric literature, see e.g. [3, 4], is sufficient. Note that the spline space $\mathbb {S}_{p,m}^2(\varGamma )$ is of dimension $n\cdot (2^m + p )^2$, where *n* denotes the number of patches involved in the description of the geometry. For the purposes of discretizing $\mathcal {W}$, we also require the space

13 $$\begin{aligned}\mathbb {S}_{p,m}^0(\varGamma ) \mathrel {\mathrel {\mathop :}=}\Big \{& f\in H^{1/2}(\varGamma ):f_{|\varGamma _j} \equiv \iota _j^{-1}(g)\\&\text { for some }g\in S_{p,p}(\varXi _{p,m},\varXi _{p,m})\Big \}, \end{aligned}$$

see, e.g., also [3, 4]. Note that that $\mathbb {S}_{p,m}^0(\varGamma )\subset \mathbb {S}_{p,m}^2(\varGamma )$ consists of globally continuous B-splines whereas $\mathbb {S}_{p,m}^2(\varGamma )$ is discontinuous across patch boundaries.

## Discretization

### Galerkin method

With the boundary integral equations and a collection of spline spaces available, we are now in the position to discretize (6) and (8). We consider a Galerkin discretization in the $L^2(\varGamma )$-duality product with the spline spaces $\mathbb {S}_{p,m}^2(\varGamma )$ and $\mathbb {S}_{p,m}^0(\varGamma )$ as ansatz and test spaces. Thus, the discrete variational formulation for (6) reads

$$\begin{aligned}&\text {Find}~t_h\in \mathbb {S}_{p,m}^2(\varGamma )~\text {such that}\\&\quad \frac{1}{2}\langle t_h,v_h\rangle _{\varGamma }+\langle \mathcal {K}^\star t_h,v_h\rangle _{\varGamma }-i\eta \langle \mathcal {V}t_h,v_h\rangle _{\varGamma }\\&\qquad =\Big \langle \frac{\partial u_{\text {inc}}}{\partial {\varvec{n}}}-i\eta u_{\text {inc}},v_h\Big \rangle _{\varGamma }\\&\quad \text {for all}~v_h\in \mathbb {S}_{p,m}^2(\varGamma ), \end{aligned}$$

with the Galerkin approximation $t_h\approx \partial u/\partial \textbf{n}$. Choosing a basis $\mathbb {S}_{p,m}^2(\varGamma )={\text {span}} \{\psi _{2,1},\ldots ,\psi _{2,N}\}$ leads to the system of linear equations

14 $$\begin{aligned} \bigg (\frac{1}{2}{} \textbf{M}_2+\textbf{K}_2^\star -i\eta \textbf{V}_2\bigg )\textbf{t}=\textbf{u}_2 \end{aligned}$$

with

$$\begin{aligned} \textbf{M}_2&=\big [\langle \psi _{2,j},\psi _{2,i}\rangle _{\varGamma }\big ]_{i,j=1}^N,\\ \textbf{K}_2^{\star }&=\big [\langle \mathcal {K}^\star \psi _{2,j},\psi _{2,i}\rangle _{\varGamma }\big ]_{i,j=1}^N,\\ \textbf{V}_2&=\big [\langle \mathcal {V}\psi _{2,j},\psi _{2,i}\rangle _{\varGamma }\big ]_{i,j=1}^N,\\ \textbf{u}_2&=\Big [\Big \langle \frac{\partial u_{\text {inc}}}{\partial {\varvec{n}}} -i\eta u_{\text {inc}},\psi _{2,i}\Big \rangle _{\varGamma }\Big ]_{i=0}^N, \end{aligned}$$

and $\textbf{t}$ being the coefficient vector of $t_h$.

The discrete variational formulation for (8) reads

$$\begin{aligned}&\text {Find}~g_h\in \mathbb {S}_{p,m}^0(\varGamma )~\text {such that}\\&\quad\frac{1}{2}\langle g_h,v_h\rangle _{\varGamma }-\langle \mathcal {K} g_h,v_h\rangle _{\varGamma }+i\eta \langle \mathcal {W}g_h,v_h\rangle _{\varGamma }\\&\qquad =\Big \langle u_{\text {inc}}-i\eta \frac{\partial u_{\text {inc}}}{\partial \varvec{n}},v_h\Big \rangle _{\varGamma }\\&\quad \text {for all}~v_h\in \mathbb {S}_{p,m}^0(\varGamma ), \end{aligned}$$

with the Galerkin approximation $g_h\approx u|_\varGamma$. Choosing a basis $\mathbb {S}_{p,m}^0(\varGamma )={\text {span}}\{\psi _{0,1}, \ldots ,\psi _{0,M}\}$ leads to the linear system of equations

15 $$\begin{aligned} \bigg (\frac{1}{2}{} \textbf{M}_0-\textbf{K}_0+i\eta \textbf{W}_0\bigg )\textbf{g}=\textbf{v}_0 \end{aligned}$$

with

$$\begin{aligned} \textbf{M}_0&=\big [\langle \psi _{0,j},\psi _{0,i}\rangle _{\varGamma }\big ]_{i,j=1}^M,\\ \textbf{K}_0&=\big [\langle \mathcal {K}\psi _{0,j},\psi _{0,i}\rangle _{\varGamma }\big ]_{i,j=1}^M,\\ \textbf{W}_0&=\big [\langle \mathcal {W}\psi _{0,j},\psi _{0,i}\rangle _{\varGamma }\big ]_{i,j=1}^M,\\ \textbf{v}_0&=\bigg [\bigg \langle u_{\text {inc}}-i\eta \frac{\partial u_{\text {inc}}}{\partial \varvec{n}},\psi _{0,i}\bigg \rangle _{\varGamma }\bigg ]_{i=0}^M, \end{aligned}$$

and $\textbf{g}$ being the coefficient vector of $g_h$.

It is well known that the matrices $\textbf{V}_2$, $\textbf{K}_0$, $\textbf{K}_2^\star$, and $\textbf{W}_0$ are dense, which makes the assembly and storage of these matrices as well as the solution of the corresponding linear systems of equations computationally prohibitively expensive for higher resolution of the ansatz spaces, i.e., large *M* or *N*. This is why we shall apply the multipole method presented in Subsection 4.4.

### Reformulation on the reference domain

Due to the isogeometric representations of the geometry, the bilinear forms for the computation of the matrix entries can entirely be pulled back to the reference domain [20]. To this end, let $\mathcal {A}$ with

16 $$\begin{aligned} (\mathcal {A}\mu )({\varvec{x}})= \int _\varGamma k({\varvec{x}},{\varvec{y}})\mu ({\varvec{y}}){\text {d}}\sigma _{\varvec{y}},\qquad {\varvec{x}}\in \varGamma, \end{aligned}$$

be one of the operators $\mathcal {V}$, $\mathcal {K}$, or $\mathcal {K}^\star$ and $\mu ,\nu :\varGamma \rightarrow \mathbb {C}$ be functions of sufficient regularity. Defining the *surface measure* of a mapping $\textbf{F}_j$ for $\hat{\varvec{x}} = (x,y)\in [0,1]^2$ as

$$\begin{aligned} a_j (\hat{\varvec{x}})\mathrel {\mathrel {\mathop :}=}\big \Vert \partial _{x}{} \textbf{F}_j(\hat{\varvec{x}}) \times \partial _{y}{} \textbf{F}_j(\hat{\varvec{x}})\big \Vert _2, \end{aligned}$$

the bilinear forms for the matrix entries can be recast as

$$\begin{aligned} \langle \mathcal {A}\mu ,\nu \rangle _\varGamma&= \sum _{j=1}^n \langle \mathcal {A}\mu ,\nu \rangle _{\varGamma _j}\\&= \sum _{i,j=1}^n \int _{\varGamma _i}\int _{\varGamma _j}k(\textbf{x},\textbf{y}) \mu ({\varvec{x}})\nu ({\varvec{y}}){\text {d}}\sigma _{\varvec{y}}{\text {d}}\sigma _{\varvec{x}}\\&= \sum _{i,j=1}^n\int _{[0,1]^2}\int _{[0,1]^2}k\big (\textbf{F}_j(\hat{\varvec{x}}),\textbf{F}_i(\hat{\varvec{y}})\big )\\&\quad \times \mu \big (\textbf{F}_j(\hat{\varvec{x}})\big )\nu \big (\textbf{F}_i(\hat{\varvec{y}})\big )a_{j}(\hat{\varvec{x}})a_{i}(\hat{\varvec{y}}){\text {d}}\hat{\varvec{y}}{\text {d}}\hat{\varvec{x}}\\&= \sum _{i,j=1}^n\int _{[0,1]^2}\int _{[0,1]^2}k_{j,i}(\hat{\varvec{x}},\hat{\varvec{y}})\mu _j(\hat{\varvec{x}})\nu _i(\hat{\varvec{y}}){\text {d}}\hat{\varvec{y}}{\text {d}}\hat{\varvec{x}}, \end{aligned}$$

with the pull-back of the kernel function and the ansatz and test functions

17 $$\begin{aligned} \begin{aligned} k_{j,i}(\hat{\varvec{x}},\hat{\varvec{y}})&=a_{j}(\hat{\varvec{x}})a_{i}(\hat{\varvec{y}})k\big (\textbf{F}_j(\hat{\varvec{x}}),\textbf{F}_i(\hat{\varvec{y}})\big ),\\ \mu _j(\hat{\varvec{x}})&=\iota _j(\mu )(\hat{\varvec{x}}),\\ \nu _i(\hat{\varvec{y}})&=\iota _i(\nu )(\hat{\varvec{y}}). \end{aligned} \end{aligned}$$

Applying a similar reasoning to the right-hand side yields

$$\begin{aligned} \langle g,\nu \rangle _\varGamma = \sum _{i=1}^n \int _{[0,1]^2} g\big (\textbf{F}_i(\hat{\varvec{x}})\big )\nu _i(\hat{\varvec{x}})a_{i}(\hat{\varvec{x}}){\text {d}}\hat{\varvec{x}}. \end{aligned}$$

Due to the additional derivative, the hypersingular operator $\mathcal {W}$ requires a special treatment which we will elaborate next.

### Regularization of the Helmholtz hypersingular operator

The hypersingular operator $\mathcal {W}$ from (5) does not have a well defined integral operator representation as in (16). Instead, it is common knowledge that the operator can be replaced by a regularized one in case of a Galerkin discretization. Namely, for the computation of the matrix entries, the representation

$$\begin{aligned}\langle \mathcal {W}\psi _{0,j},\psi _{0,i}\rangle _{\varGamma } ={}&\langle \mathcal {V}{\text {curl}}_{\varGamma }\psi _{0,j},{\text {curl}}_{\varGamma }\psi _{0,i}\rangle _{\varGamma }\\&-\kappa ^2\int _{\varGamma }\int _{\varGamma }G({\varvec{x}},{\varvec{y}}) \langle {\varvec{n}}_{\varvec{x}},{\varvec{n}}_{\varvec{y}}\rangle _{\mathbb {R}^3} \psi _{0,j}({\varvec{x}})\psi _{0,i}({\varvec{y}}){\text {d}}\sigma _{\varvec{y}}{\text {d}}\sigma _{\varvec{x}}, \end{aligned}$$

$i,j=1,\ldots ,M$, can be used, see e.g. [26]. Therein, ${\text {curl}}_{\varGamma }\psi _{0,i}$ denotes the surface curl which maps a scalar valued function on the surface into a vector field in the tangential space of $\varGamma$. On any given patch $\varGamma _j$, the isogeometric representations of the boundary of the scatterer allow for its explicit representation

18 $$\begin{aligned} \begin{aligned} {\text {curl}}_{\varGamma }\psi _{0,i}(\textbf{x})&=\frac{1}{a_i(\hat{\textbf{x}})} \Big (\partial _{\hat{x}_1}\iota _j(\psi _{0,i})(\hat{\textbf{x}}) \partial _{\hat{x}_2}{} \textbf{F}_j(\hat{\textbf{x}})\\&\quad -\partial _{\hat{x}_2}\iota _j(\psi _{0,i})(\hat{\textbf{x}}) \partial _{\hat{x}_1}{} \textbf{F}_j(\hat{\textbf{x}})\Big ) \end{aligned} \end{aligned}$$

for all $\textbf{x}=\textbf{F} _j(\hat{\textbf{x}})\in \varGamma _j$, $\hat{\textbf{x}}\in [0,1]^2$, see [14] for example for the precise derivation. This amounts to the following expression of the hypersingular operator in closed form

19 $$\begin{aligned}\langle \mathcal {W}\psi _{0,k},\psi _{0,\ell }\rangle _{\varGamma } &= \sum _{i,j=1}^n\int _{[0,1]^2}\int _{[0,1]^2}k_{j,i}(\hat{\varvec{x}},\hat{\varvec{y}})\\&\quad \times \bigg ( \nabla _{\hat{\varvec{x}}}\iota _j(\psi _{0,k})(\hat{\varvec{x}})^\intercal K_{j,i}(\hat{\varvec{x}},\hat{\varvec{y}})^{-1} \nabla _{\hat{\varvec{y}}}\iota _i(\psi _{0,\ell })(\hat{\varvec{y}}) \\&\quad\qquad -\kappa ^2 \langle {\varvec{n}}_{\varvec{x}},{\varvec{n}}_{\varvec{y}}\rangle _{\mathbb {R}^3}\iota _j(\psi _{0,k}) (\hat{\varvec{x}})\iota _i(\psi _{0,\ell })(\hat{\varvec{y}})\bigg ){\text {d}}\hat{\varvec{y}}{\text {d}}\hat{\varvec{x}}, \end{aligned}$$

where the pull-back of the kernel $k_{j,i}$ is given by

$$\begin{aligned} k_{j,i}(\hat{\varvec{x}},\hat{\varvec{y}})=k\big (\textbf{F}_j(\hat{\varvec{x}}),\textbf{F}_i(\hat{\varvec{y}})\big ) \end{aligned}$$

and $K_{j,i}$ denotes the first fundamental tensor of differential geometry,

$$\begin{aligned} K_{j,i}(\hat{\varvec{x}},\hat{\varvec{y}}) = \big [ \langle \partial _{\hat{\varvec{x}}_k}{} \textbf{F}_j(\hat{\varvec{x}}), \partial _{\hat{\varvec{x}}_l}{} \textbf{F}_i(\hat{\varvec{y}})\rangle _{\mathbb {R}^3}\big ]_{k,l=1}^2 \in \mathbb {R}^{2\times 2}. \end{aligned}$$

Compared to the Laplace case, see [14], we note the occurrence of a second term in the regularized representation (19). However, this additional term behaves similar to the single layer operator and thus poses no further challenges for the implementation.

For the numerical evaluation of the first term in (19), recall that an ansatz function $\psi _{0,j}|_{\varGamma _i}$ on the patch $\varGamma _i$ is given by $\psi _{0,j} = \iota _i^{-1}(\hat{\psi })$ for some $\hat{\psi }\in S_{p,p}(\varXi _{p,m},\varXi _{p,m})$, see (13). There therefore holds

$$\begin{aligned} \nabla _{\hat{\varvec{x}}}\iota _i(\psi _{0,i})(\hat{\varvec{x}}) = \nabla _{\hat{\varvec{x}}}\iota _i\big (\iota _i^{-1}(\hat{\psi })\big )(\hat{\varvec{x}}) = \nabla _{\hat{\varvec{x}}}\hat{\psi }(\hat{\varvec{x}}). \end{aligned}$$

Thus, for each basis function $\varphi \otimes \psi \in S_{p,p}(\varXi _{p,m},\varXi _{p,m})$, one has only to provide its derivatives $\varphi '\otimes \psi$ and $\varphi \otimes \psi '$. These derivatives, however, are readily available in implementations and they belong to the spline spaces

$$\begin{aligned} \partial _1S_{p,p}(\varXi _{p,m},\varXi _{p,m})&=S_{p-1,p}(\varXi _{p,m}',\varXi _{p,m}),\\ \partial _2S_{p,p}(\varXi _{p,m},\varXi _{p,m})&=S_{p,p-1}(\varXi _{p,m}',\varXi _{p,m}'), \end{aligned}$$

where $\varXi _{p,m}'$ denotes the truncation of $\varXi _{p,m}$, i.e., the knot vector $\varXi _{p,m}$ without its first and last knot.

### Fast multipole method

The black-box fast multipole method, see [18], relies on a degenerate kernel approximation of the integral kernel under consideration. Such an approximation is available in the kernel’s far-field, which means that the supports of the trial and test functions have to be sufficiently distant from each other—they are *admissible*.

One arrives at an efficient algorithm, if one subdivides the set of trial functions hierarchically into so-called clusters. Then, the kernel interaction of two clusters is computed by using the degenerate kernel approximation if the clusters are admissible. This means a huge matrix block in the system matrix is replaced by a low-rank matrix. If the clusters are not admissible, then one subdivides them and considers the interactions of the respective children. That way, the assembly of the Galerkin matrix can be performed in essentially linear complexity, given that the parametrization of each patch is smooth.

For the realization of the multipole method in the present context of isogeometric boundary element methods, we refer the reader to [13, 14]. A particular advantage of the referred compression method is that the isogeometric setting allows to perform the compression of the system matrix in the reference domain rather than the computational domain. This means that we consider the pull-back of the kernel (17) instead of the kernel in free space, as originally proposed in [20], while the admissibility is still applied in the physical space. As a consequence, the rank of the low-rank blocks in the number of one-dimensional interpolation points *p* decreases from $\mathcal {O}(p^3)$ to $\mathcal {O}(p^2)$. The compressed matrix is finally represented in the $\mathcal {H}^2$-matrix format as usual, see [2].

For the potential evaluation, i.e., for evaluating (7) and (9), we exploit a similar approximation of the kernel function. However, this time we perform the low-rank approximation in physical space, that is, we employ a degenerate kernel approximation for the kernel $k$. Rather than clustering elements as before, we directly cluster evaluation and quadrature points and realize the potential evaluations by means of matrix-vector multiplications. The rank of the low-rank blocks is in this case $\mathcal {O}(p^3)$. In particular, we may employ a matrix-free version, as all blocks are only required once. The advantage of this approach becomes immanent if the number of potential evaluation points increases proportionally to the number of degrees of freedom in the linear system of equations. In this case, the cost of the proposed potential evaluations scales essentially linearly instead of quadratically.

## Numerical experiments

### Setup

The numerical experiments are performed by using the publicly available C++ library Bembel, see [10, 11]. To this end, the previously not available operators (double layer, adjoint double layer, and hypersingular operator) were implemented. Each of the matrices in the combined field integral equations (14) and (15) was computed separately in compressed form as $\mathcal {H}^2$-matrix by using the fast multipole method on the reference domain as described in [11, 14]. The compression parameters for the fast multipole method were set to the default values ($\eta =1.6$, nine interpolation points per direction), see [11, 14] for more details. The product of the matrix sums with vectors was implemented using lazy evaluation and the arising linear systems of equations (14) and (15) were solved up to relative machine precision by means of a restarted GMRES method with a restart after 30 iterations. Finally, all computations were performed in parallel by using the built-in OpenMP-parallelization of Bembel on a compute server with 1.3 terabyte RAM and four Intel(R) Xeon(R) E7-4850 v2 CPU with twelve 2.30GHz cores each and hyperthreading disabled.

### Convergence benchmark

**Fig. 2:**
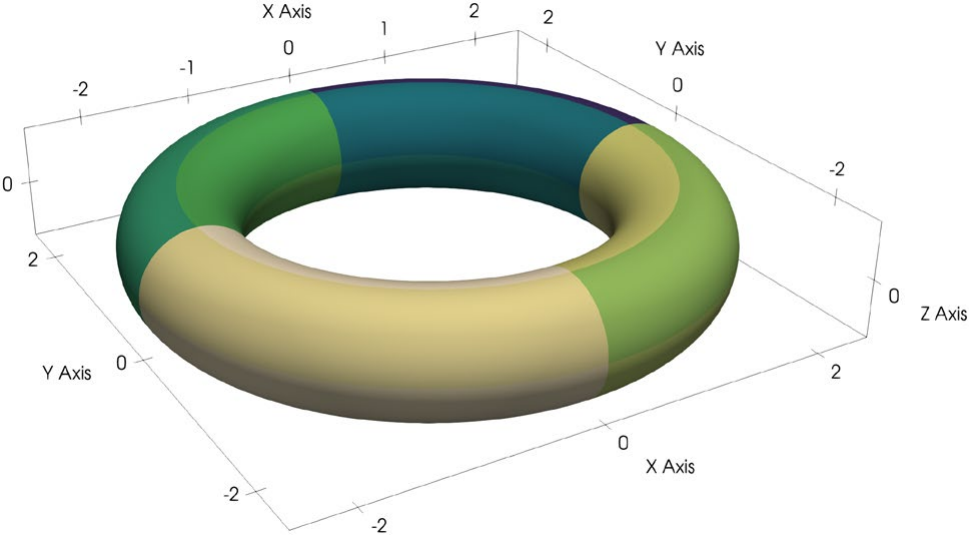
Torus represented by 16 patches and illustration of its dimensions

**Fig. 3:**
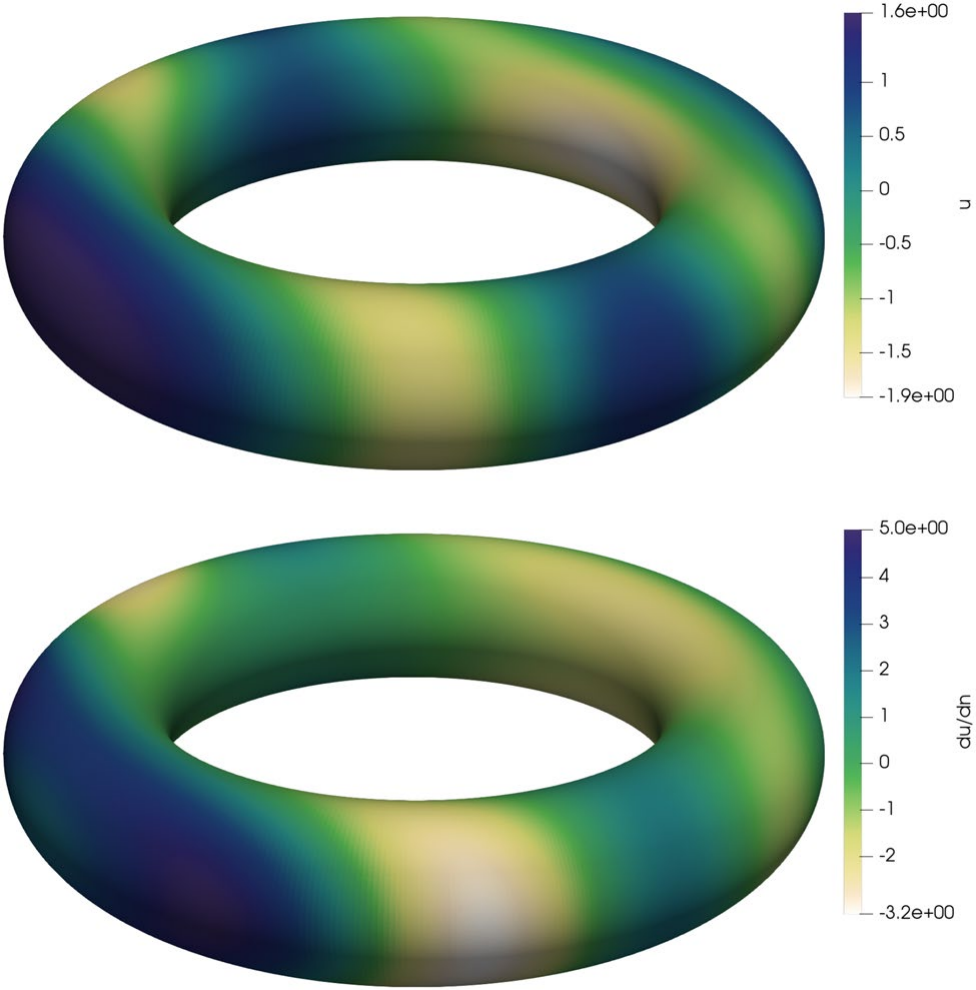
The Dirichlet data *u* (top) of the total wave in case of a sound-hard torus and the Neumann data $\partial u/\partial {\varvec{n}}$ (bottom) of the total wave in case of a sound-soft torus

In order to study convergence rates, we consider a torus with major radius two and minor radius 0.5 that is represented by 16 patches, see Fig. 2 for an illustration. On this geometry, we aim at computing the scattered wave of a plane incident wave in *x* direction with wavenumber 2.5. The scattered wave is then measured on 100 points distributed on a sphere with radius 5 around the origin. We refer to Fig. 3 for an illustration of the Dirichlet data of the total wave (top plot) in case of a sound-hard torus and the Neumann data of the total wave (bottom plot) in case of a sound-soft torus.

**Fig. 4:**
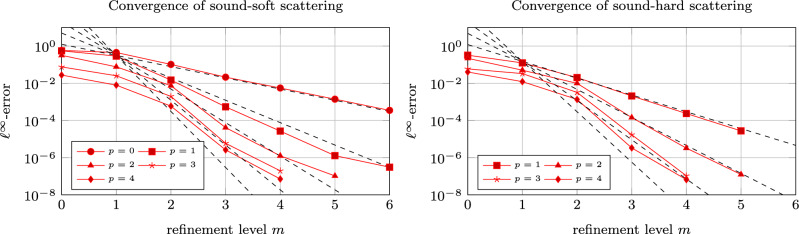
Convergence of the combined field integral equations for various polynomial degrees. The dashed lines illustrate the expected convergence rates of $\mathcal {O}\big (h^{2p+2}\big )$ in case of sound-soft obstacles (left) and $\mathcal {O}\big (h^{2p+1}\big )$ in case of sound-hard obstacles (right)

The optimal convergence rates for the potential evaluation in case of splines of degree *p* are $\mathcal {O}\big (h^{2p+2}\big )$ for the boundary integral equation (6) which corresponds to sound-soft obstacles and $\mathcal {O}\big (h^{2p+1}\big )$ for the boundary integral equation (8) which corresponds to sound-hard obstacles. Since the obstacle under consideration is smooth, we should achieve these convergence rates. Note that these rates are twice as high as for the collocation method and are known as the *super convergence* of the Galerkin formulation, see [30] for example. Figure 4 validates that we indeed reach the theoretical convergence rates, up to the consistency error induced by the far-field interpolation of the fast multipole method, which causes the stagnation of the error at around $10^{-6}$. For a systematic study of this consistency error, we refer to [14]. As a reference, we consider here the solutions obtained from an indirect formulation using a single layer or adjoint double layer ansatz, respectively.

**Fig. 5:**
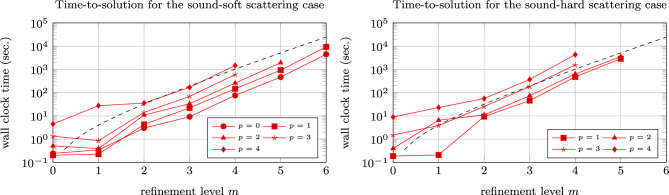
Scaling of the combined field integral equations for various polynomial degrees. The dashed lines illustrate log-linear scaling

Figure 5 illustrates the scaling of the runtimes of the computations. Instead of a quadratic scaling of the runtimes, which we would have in the case of a traditional boundary element method, one figures out that the multipole-accelerated isogeometric boundary element method scales essentially linearly as expected. This enables large-scale calculations as we will consider in the next example.

### Computational benchmark

**Fig. 6:**
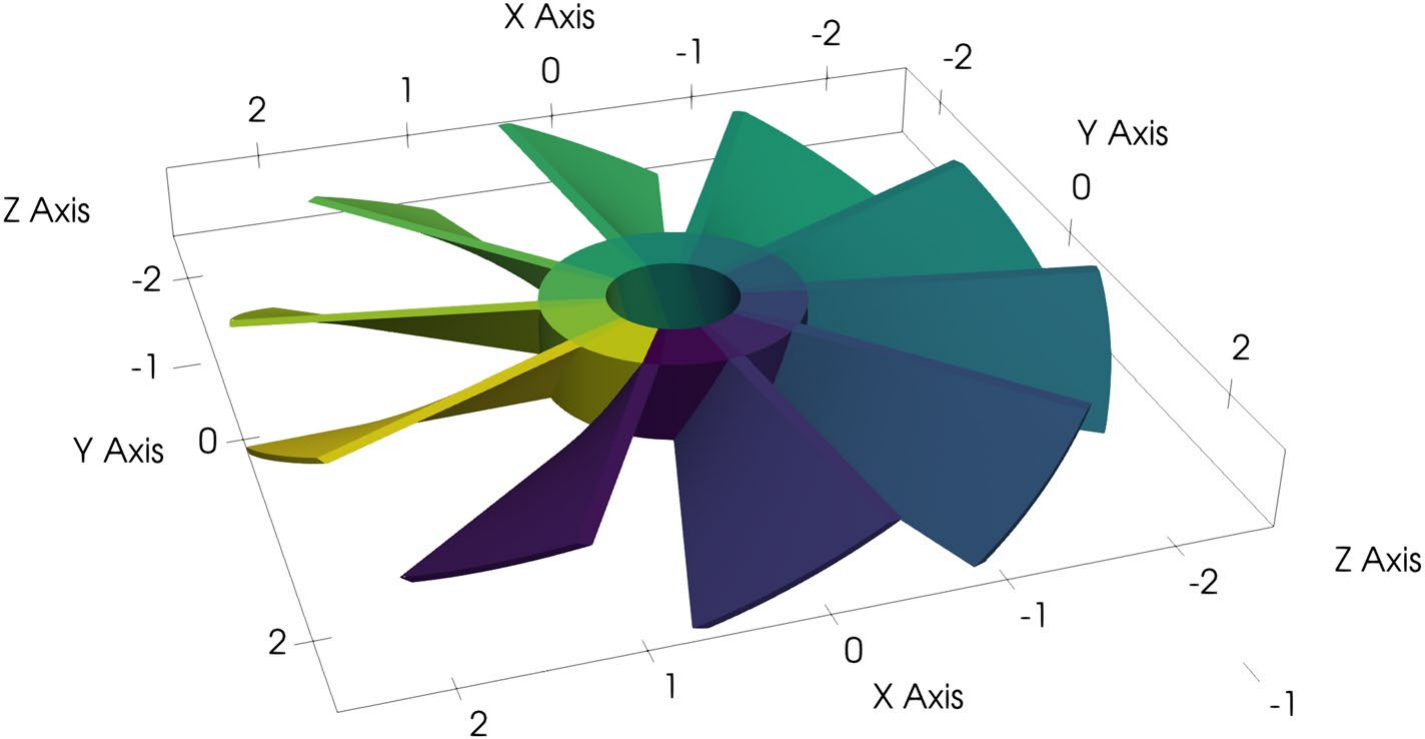
Turbine geometry with 120 patches

As a computational benchmark, we consider a turbine with ten blades that is parametrized by 120 patches as illustrated in Fig. 6. Thereof, it can be figured out that the turbine has a diameter of 5. Again, we compute the scattered wave of a plane incident wave in *x* direction, but with wavenumber 1.0.

We choose cubic B-splines and three refinement levels to discretize the Cauchy data *u* and $\partial u/\partial {\varvec{n}}$ on the surface geometry. This results in 14,520 degrees of freedom in case of a sound-soft turbine and 12,000 degrees of freedom in case of a sound-hard turbine, respectively. The overall solution time for assembly and solution of the underlying systems of linear equations requires only about a few hours.

**Fig. 7:**
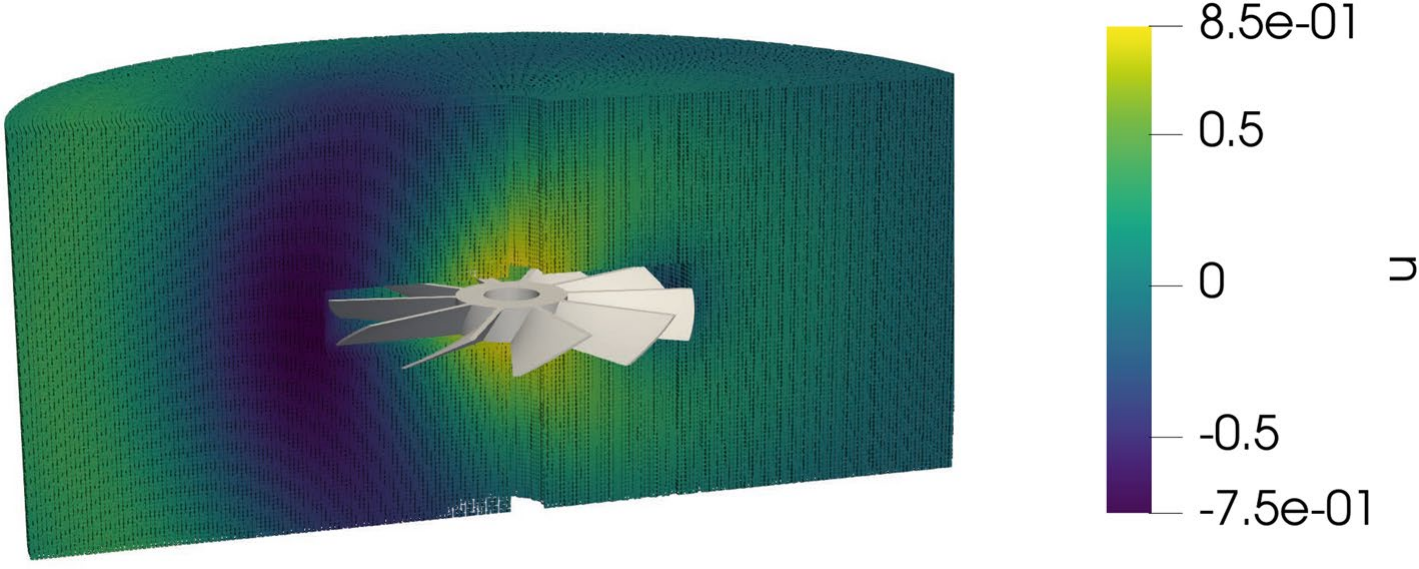
Scattered wave evaluated in 3,664,832 points for the sound-hard case

**Fig. 8:**
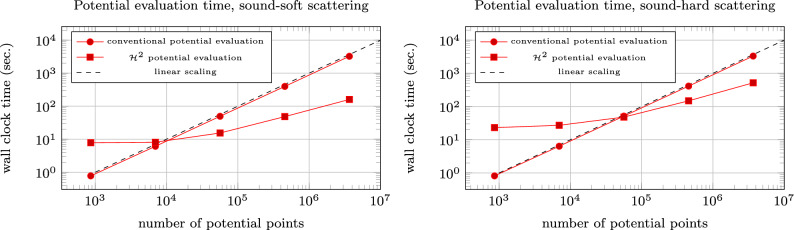
Computation time of conventional and $\mathcal {H}^2$-matrix accelerated potential evaluation for various number of points

We compute next the scattered wave in a cylinder on up to 3,664,832 points, see Fig. 7 for an illustration. To demonstrate the efficiency of the fast potential evaluation, we compare the scaling of the multipole-accelerated potential evaluations with the traditional potential evaluations. Figure 8 illustrates that—after a certain warm-up phase for only a few potential points—the $\mathcal {H}^2$-matrix accelerated potential evaluation is indeed superior to the conventional one when increasing the number of evaluation points. Consequently, the calculation of the scattered wave also in free space becomes feasible and very efficient.

## Conclusion

In this article, we have discussed an isogeometric, frequency stable algorithm for the solution of acoustic obstacle scattering problems with essentially linear complexity in the number of boundary elements and potential evaluation points. The algorithm itself is based on a boundary reduction of the problem by means of combined field integral equations which are dealt with isogeometrically. The integral equations are discretized by the Galerkin method, for which an appropriate regularization of the hypersingular operator is available such that it fits into the isogeomreic framework. To deal with the non-locality of integral equations and potential evaluation, we employed two versions of the FMM. For the boundary integral equations, all dense system matrices have been compressed with the isogeometric embedded FMM. For the potential evaluation in space, we employed an FMM in space. We have presented convergence benchmarks that demonstrate the high accuracy of the isogeometric boundary element method. In addition, we have considered a complex computational benchmark on a complex geometry, which corroborates the feasibility of the approach in the engineering practice.

## Footnotes

1 www.bembel.eu.
